# Tilted Fiber Bragg Grating Sensor with Graphene Oxide Coating for Humidity Sensing

**DOI:** 10.3390/s17092129

**Published:** 2017-09-15

**Authors:** Yung-Da Chiu, Chao-Wei Wu, Chia-Chin Chiang

**Affiliations:** 1 Department of Mechanical Engineering, National Kaohsiung University of Applied Sciences, No. 415, Jiangong Rd., Sanmin Dist., Kaohsiung 807, Taiwan; andy830130@gmail.com; 2 Department of Aeronautical and Mechanical Engineering, Air Force Academy, Kaohsiung 807, Taiwan; cafa95011@gmail.com

**Keywords:** humidity sensor, tilted fiber Bragg grating, graphene oxide

## Abstract

In this study, we propose a tilted fiber Bragg grating (TFBG) humidity sensor fabricated using the phase mask method to produce a TFBG that was then etched with five different diameters of 20, 35, 50, 55 and 60 μm, after which piezoelectric inkjet technology was used to coat the grating with graphene oxide. According to the experimental results, the diameter of 20 μm yielded the best sensitivity. In addition, the experimental results showed that the wavelength sensitivity was −0.01 nm/%RH and the linearity was 0.996. Furthermore, the measurement results showed that when the relative humidity was increased, the refractive index of the sensor was decreased, meaning that the TFBG cladding mode spectrum wavelength was shifted. Therefore, the proposed graphene oxide film TFBG humidity sensor has good potential to be an effective relative humidity monitor.

## 1. Introduction

After it was first developed, most optical fiber was used in telecommunications applications, but in recent years the development of fiber optic sensors has proceeded rapidly, especially with respect to temperature and stress sensors. Furthermore, fiber optic sensors for biomedical and home security purposes have also been developed in recent years. The mechanism by which such sensors provide measurements is very dependent on changes in the refractive index, such as in the case of LPG gas sensor measurements of CO_2_ [1] and U-shaped bending-induced interference sensor measurements of glucose solutions [2]. However, these previously developed sensing methods have not taken into account the impact of environmental humidity. Therefore, according to the following references, optical fiber sensors can be applied to provide a variety of different physical measurements, including measurements of humidity [3,4,5,6,7,8], strain, temperature [3,4], and RI, with some sensors even being versatile enough to measure several phenomena at once [9]. The present study proposes a humidity sensor that could potentially be used to aid other sensors in adjusting to environmental humidity. It should be noted, however, that various other types of fiber optic humidity sensors have previously been reported. For example, a side-polished fiber sensor was fabricated with a wheel side-polishing technique [10], a long period fiber grating sensor was fabricated using a multi-layer dip coating technique [11], and a hollow core fiber sensor was achieved at a resonant wavelength [12]. However, the methods used to fabricate those sensors are complicated. Therefore, the advantages of the sensor proposed in this study are its low manufacturing cost and short processing time.

The proposed sensor utilizes a tilted fiber Bragg grating (TFBG) to achieve the humidity sensing function because TFBG can effectively measure the surrounding refractive index. This is because when the refractive index changes, the wavelength of the cladding mode of the TFBG will shift in a way that corresponds to the ambient humidity value.

Humidity sensors are often used in conjunction with cooled computers, heat treating furnaces, smelting furnaces, clean room controls, and dryers, as well as in applications relating to drug administration, food preservation, and climatic measurements. However, while some harsh environments, such as high temperature environments or environments with high levels of electromagnetic waves, will have an impact on the sensitivity of previously produced humidity sensors, optical fiber humidity sensors could potentially overcome those environmental challenges.

In TFBG, there is a certain tilt angle between the grating plane and the fiber, so the transmission spectrum possesses many resonances [13], resulting in the occurrence of more complex mode coupling. Core and cladding mode coupling, including coupling involving the core mode and radiation mode, also occur in TFBGs. Since the tilt angle and refractive index modulation determine the coupling efficiency and the bandwidth of cladding mode resonances [14], the transmission characteristics of TFBG provide a great amount of information related to the fiber and grating structures.

In 1996, Erdogan and Sipe [15], using a high-intensity ultraviolet radiation grating written in fiber, presented a detailed theory of how Bragg reflection and radiation mode coupling loss are combined in TFBG. In 2001, Li et al. [16] proposed a tilt-type fiber grating that uses the volumetric current (VCM) method to calculate radiation morphology (including wavelength dependence, azimuth distribution, and polarization dependence). In 2001, Feder et al. [17] used a spectrum analyzer and the radiation pattern of the core and the cladding mode of TFBG to provide multi-band Raman pump power measurements. In 2012, Wang et al. [18] introduced the sensing principles of a polyimide-coated FBG humidity sensor. The results obtained for this sensor, including a sensitivity of 2 pm/% RH and a measurement range of 30% to 80%, showed that polyimide resin is an ideal coating material for use in the manufacturing of an FBG humidity sensor. In 2013, Berruti et al. [19] proposed a polyimide-coated FBG humidity sensor with a wavelength sensitivity of 0.00213 nm/% RH in a humidity range of 0% to 75% at low temperatures. In 2014, Montero et al. [20] applied steel and carbon fiber polymers to FBG measuring a range of 30% to 90% RH in a temperature range of 10~70 °C; in the range of 30% to 80% RH, the sensitivity wavelength was 0.001375 nm/% RH. In 2016, Wang et al. [21] proposed coating graphene oxide on TFBG as a means of sensing humidity via the adsorption and desorption of water by the graphene oxide film. With the use of cladding mode and graphene oxide film interaction between the detection wavelength at 1557 nm and the transmission loss change, a maximum sensitivity of 0.129 dB/% RH with a linear correlation coefficient in the humidity range of 10% to 80% and 99% was achieved, indicating that this humidity sensor has repetitive stability and other good characteristics. According to the above literature, the humidity measurement range of such sensors is about 30% to 80% RH, with the transmission loss changes yielding an average sensitivity of 0.00168 nm/% RH. As these sensors are made using the arc fusion splicing method, where the sensing layer has a hydrogel coating, the above method is highly time-consuming and involves a high degree of complexity.

Therefore, this study proposes for the first time, to the best of our knowledge, a small diameter TFBG sensor (20 μm) with a graphene oxide coating for humidity sensing. The novelty of the proposed TFBG sensor is twofold: first, the sensitivity of the sensor is increased by reducing the fiber diameters via etching; second, we provide new coating technology used the piezoelectric inkjet can provide a uniform graphene oxide coating and fast processing, allowing it to be used as a humidity sensor.

## 2. Working Principle of the Graphene Oxide Humidity Sensor

**Figure 1:**
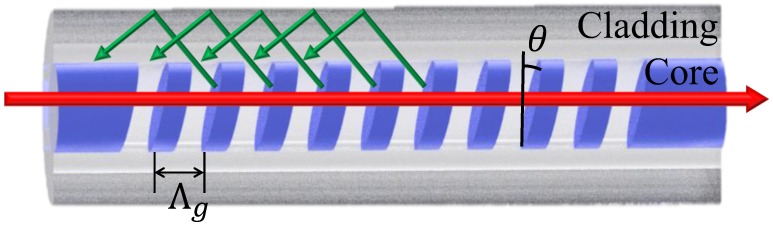
Schematic diagram of TFBG.

Mode fiber coupling between the wave vectors of all vector fields occurs along the fiber axis to form a uniform axial conduction mode structure that is defined by a flat phase plane perpendicular to the guide shaft, as shown in Figure 1. The resonant wavelength satisfying the Bragg condition for an ordinary fiber grating can be expressed by the following Equation [14]:

(1) $$\lambda_{Bragg}=\left(n_{eff,core}+n_{eff,core}\right)\Lambda_{g}$$

The Bragg resonance conditions described above are produced by the coupling of mode-forward propagating core modes and back-propagating core modes. For TFBG, the inclination angle of the grating surface and the axis of the fiber, as well as the grating period along the fiber axis, can be modified according to the following Equation [14]:

(2) $$\Lambda_{g}=\frac{\Lambda }{\cos\theta }$$

Substituting Equation (2) into Equation (1), yields the following:

(3) $$\lambda_{Bragg}=\left(n_{eff}^{co}+n_{eff}^{co}\right)\frac{\Lambda }{\cos\theta }$$

Due to the presence of the tilted angle, part of the light propagating in a forward direction through the core mode will be coupled to the cladding mode of the backward propagation, and the resonant wavelength of the cladding mode will be determined by the following equation [14]:

(4) $$\lambda_{Cl,i}=\left(n_{\mathrm{eff}}^{\mathrm{co}}+n_{\mathrm{eff},i}^{\mathrm{cl}}\right)\frac{\Lambda }{\cos\theta }$$

where $n_{\mathrm{eff},i}^{\mathrm{cl}}$ is the effective refractive index of the ith cladding mode. As the effective refractive index increases, the wavelength redshifts, and from Equation (2), it can be seen that using a fixed grating period to change the rotation angle can change the Bragg period. The purpose of this study was to investigate the cladding mode wavelength shift, and by inserting the measurement data into Equation (4), we could verify whether the measurements provided by the TFBG humidity sensor were correct.

Due to the interaction between the cladding mode and the graphene oxide, the resonance of the cladding mode changes as the graphene oxide adsorption process proceeds, because the effective refractive index of the graphene oxide film changes as the water molecules are adsorbed. As can be seen from Equation (4), when the humidity increases, the refractive index changes, causing the wavelength to be shifted, and through this phenomenon, the changes in the TFBG sensor effectively correspond to the relative humidity changes.

## 3. Experiment

### 3.1. Processing and Fabrication of TFBG

**Figure 2:**
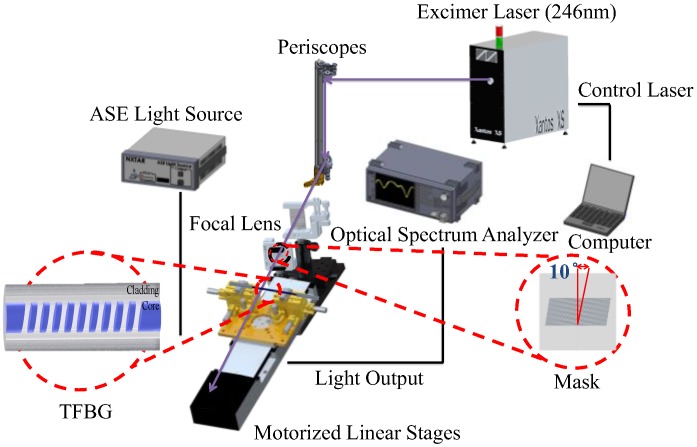
Processing and fabrication of TFBG sensor.

In this study, the TFBG humidity sensor was fabricated using single-mode fiber (SMF-28), the phase mask method, and piezoelectric inkjet technology. Using a wire stripper, 3 cm of the protective middle layer was stripped from optical fiber, after which the fiber was wiped clean with alcohol and then placed on a 3-axis micro-platform. Next, the mask platform was rotated 10 degrees and excimer laser (Xantos XS, Excimer laser, COHERENT, Lübeck, Germany) irradiation was applied through phase masks (1075.5 nm, Phase mask, Ibsen photonics, Farum, Denmark) to produce the grating formation. An optical spectrum analyzer was then utilized to confirm the generation of TFBG in the core of the spectrum structure, as shown in Figure 2. The length of the TFBG was 5 mm. The TFBG was processed using buffered oxide etch (BOE) to change the diameter of the fiber by etching. Approximately 3 cm of the protective layer at the center of a single-mode fiber section was removed, and the fiber was adhered to the etching board, which was then placed into a plastic box into which BOE was poured to effect the etching.

**Figure 3:**
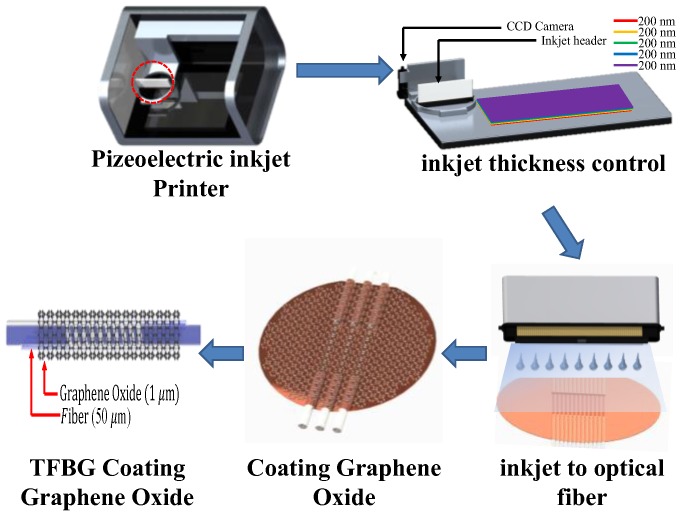
Flow chart of the process of using piezoelectric inkjet technology to coat the fiber with graphene oxide.

The TFBG was etched to 20 μm, and then piezoelectric inkjet technology (DMP-2850, Dimatix Materials Printer, FUJIFILM Value From Innovation, Santa Clara, CA, USA) was used to spray an aqueous solution of graphene oxide onto the surface of the optical fiber. The coated fiber was then heated to 100 degrees on a baking plate to induce evaporation, allowing the graphene to become more securely attached to the optical fiber surface, as shown in Figure 3.

### 3.2. The Setup of the Humidity Sensing Experiment

**Figure 4:**
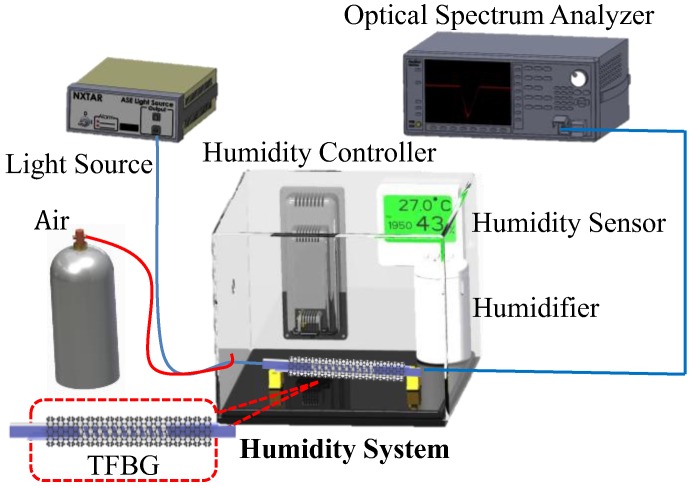
Experimental setup for humidity sensing.

The fiber was placed in a humidity control box. A light source (ASE-2200, ASE light source, NXTAR Technologies Inc., Tainan, Taiwan) was attached to one end of the box, while an optical spectrum analyzer was attached to the other. humidification control range of 20–80% RH, temperature controller setting 27 °C, standard humidity sensor for compared to our TFBG GO humidity sensor, humidifier to increase the environment relative humidity, dehumidification is the dry air into the (a flow rate of 10 L/min) humidity control Box to reduce humidity, as shown in Figure 4.

In this study, the TFBG-coated graphene oxide film coated on SMF-28 single-mode photosensitive fiber was etched with five different diameters of 20, 35, 50, 55 and 60 μm, and the relative humidity was measured at a constant temperature of 27 °C and a humidity range of 20% to 80% RH.

## 4. Results and Discussion

### 4.1. Graphene Oxide Film Coating

**Figure 5:**
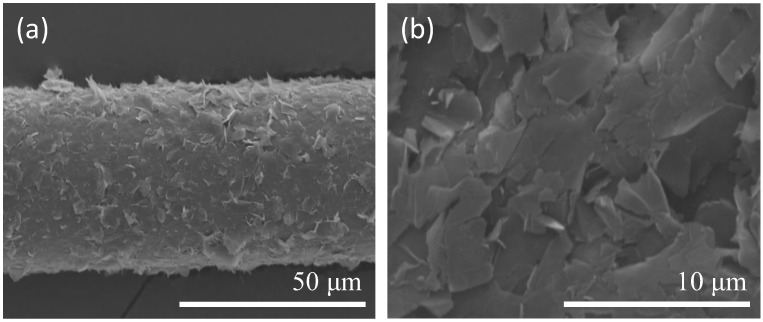

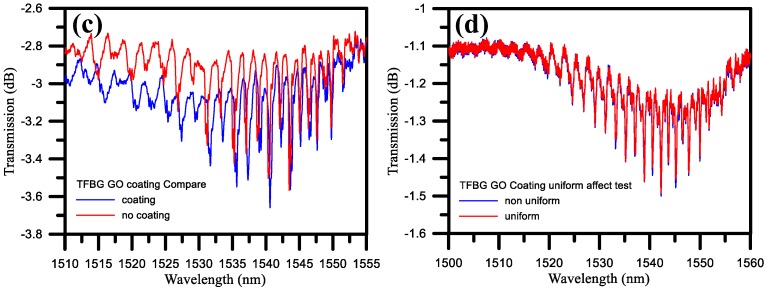
(**a**) Scanning Electron Microscope (SEM) image of TFBG coated with graphene oxide film; (**b**) SEM image of graphene oxide film; (**c**) TFBG GO coating comparison; (**d**) TFBG GO uniform coating comparison.

In this study, piezoelectric inkjet technology was used to coat graphene oxide film on optical fiber. The thickness of the coated graphene oxide, as measured by scanning electron microscope (SEM) imaging, was about 1 μm. Enlargement of the SEM image shown in Figure 5a revealed that the graphene film was in the form of a sheet, thereby proving that the graphene oxide was coated on the optical fiber sensor (Figure 5b). We sought to compare the effects of a uniform coating with those of a nonuniform coating. As shown in Figure 5c,d, the nonuniform coating resulted in a slight degree of transmission variation relative to the uniform coating, but the amount of variation was minimal and would not significantly affect the measurement results.

When the humidity in the air increases, more water molecules are adsorbed on the graphene oxide, and the water molecules on the graphene layer will, in turn, change the gap between the graphene and the fiber. In a previous study of graphene oxide thin films [22], these changes were found to be due to the bonding between the dissociated H_2_O molecules (H_2_O = H^+^ + OH^−^) and the C−OH and C=O groups of the GO. Such an increase in interlayer spacing is also consistent with previous studies on the interlayer spacing of graphene oxide as a function of relative humidity. 

### 4.2. Temperature Response of the TFBG Sensor

**Figure 6:**
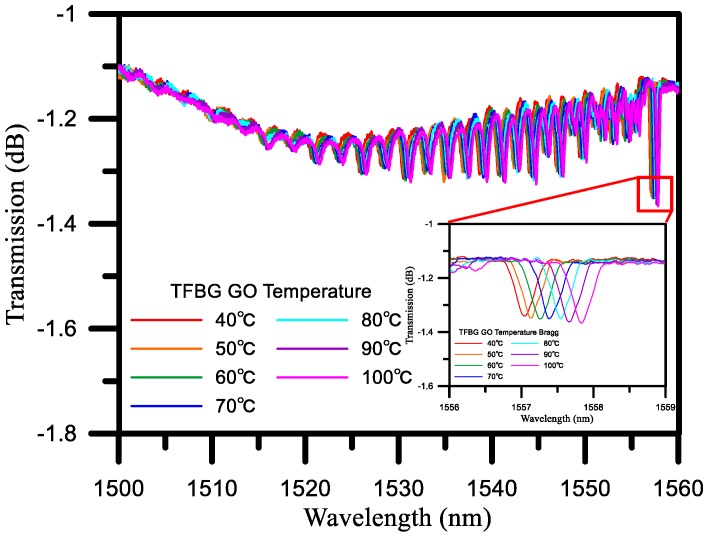
Effects of temperature on TFBG GO wavelengths.

We conducted an initial temperature experiment to verify the effect of temperature on the TFBG sensor, with the results being shown in Figure 6. As the temperature was raised from 40 °C to 100 °C, the wavelength was shifted from a short wavelength to a long wavelength, with the wavelength sensitivity being 0.014 nm/°C. This can be compensated for by Bragg mode temperature compensations. We will use temperature control by ±1 degrees C to reduce the effect of temperature.

### 4.3. Comparison of Different Diameters

**Figure 7:**
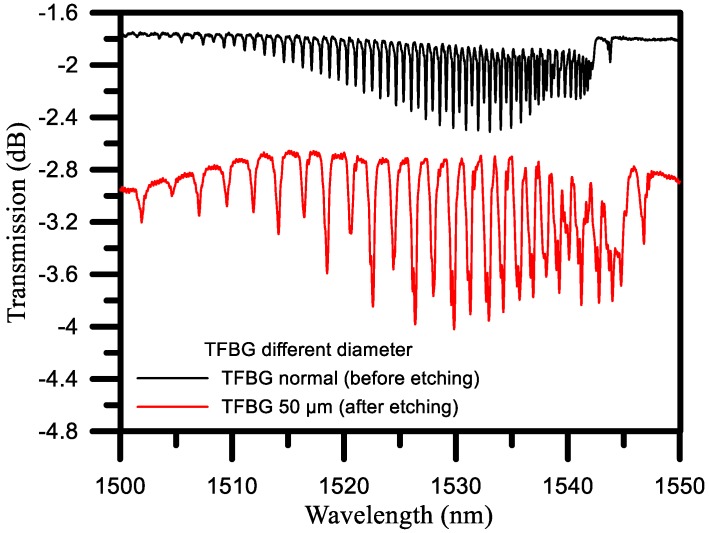
TFBG spectrum, before etching (Black line), after etching (Red line).

The strength of the transmission, which is less strong without the etching, becomes stronger after the etching, resulting in the wavelength being shifted from a short wavelength to a long wavelength. After etching, the cladding mode is significantly enhanced, which improves the ability of the fiber to measure the refraction index, as shown in Figure 7. Therefore, this study explored how different diameters of the TFBG affect its sensitivity.

The TFBG coating graphene oxide humidity sensor has the best sensitivity in the number of diameters. In this paper, the sensitivity of the sensor to humidity changes was measured with different diameters of 20, 35, 50 55 and 60 μm. As can be seen from the discussion in Section 4.1 above, when the level of water molecules in the air is increased, the molecules will, in turn, increase the gap between the graphene and the fiber, thereby changing the external refractive index of the TFBG. 

**Figure 8:**
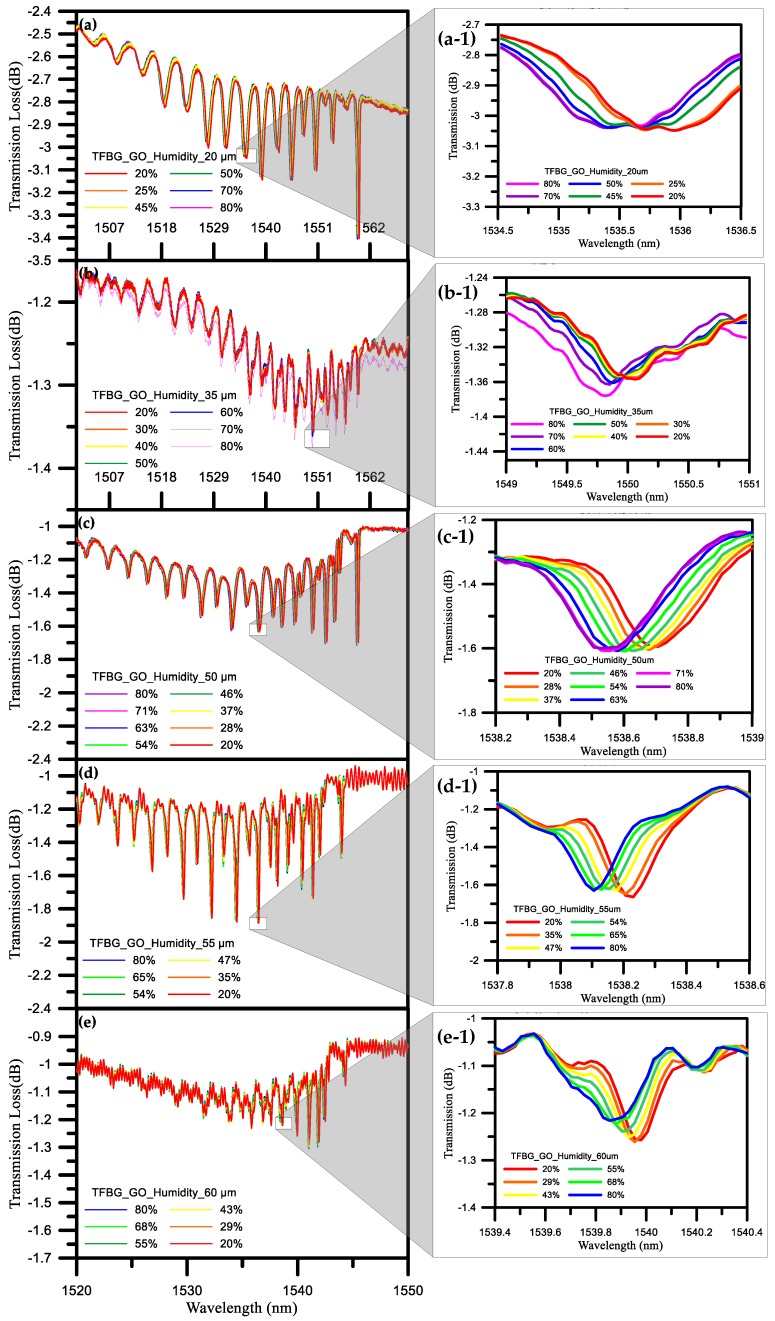
TFBG humidity sensor spectra with different diameters of (**a**) 20 μm; (**b**) 35 μm; (**c**) 50 μm; (**d**) 55 μm; and (**e**) 60 μm. Insets: (**a**-**1**) 1535.367 nm to 1535.962 nm; (**b**-**1**) 1549.815 nm to 1550.060 nm; (**c**-**1**) 1538.2 nm to 1539 nm; (**d**-**1**) 1537.8 nm to 1538.6 nm; and (**e**-**1**) 1539.4 nm to 1540.4 nm.

As shown in Figure 8a, when the diameter was 20 μm and the RH was at 20%, the wavelength was 1535.962 nm, whereas the wavelength was 1535.367 nm at an RH of 80%, meaning that the shift of the wavelength was 595 pm. As shown in Figure 8b, when the diameter was 35 μm and the RH was 20%, the wavelength was 1550.060 nm, whereas the wavelength was 1549.815 nm at an RH of 80%, meaning that the shift of the wavelength was 245 pm. As shown in Figure 8c, when the diameter was 50 μm and the RH was 20%, the wavelength was 1538.706 nm, whereas the wavelength was 1538.531 nm at an RH of 80%, meaning that the shift of the wavelength was 175 pm. As shown in Figure 8d, when the diameter was 55 μm and the RH was 20%, the wavelength was 1538.231 nm, whereas the wavelength was 1538.106 nm at an RH of 80%, meaning that the shift of the wavelength was 125 pm. Finally, as shown in Figure 8e, when the diameter was 60 μm and the RH was 20%, the wavelength was 1539.980 nm, whereas the wavelength was 1539.855 nm when the RH was 80%, meaning that the shift of the whole wavelength was 125 pm.

**Figure 9:**
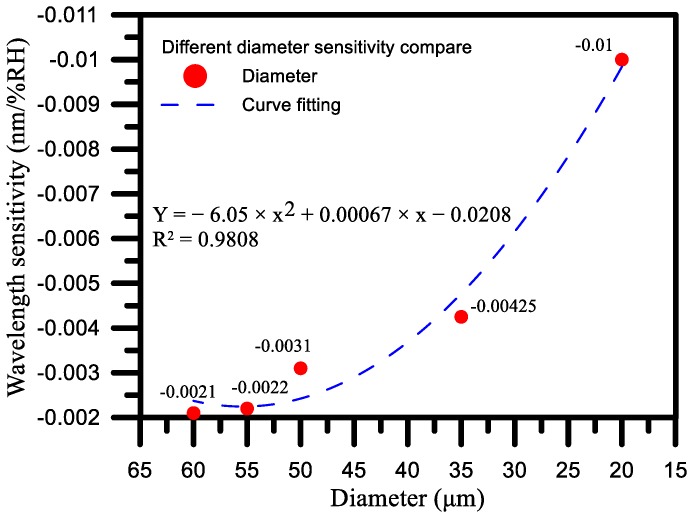
Comparison of sensitivity of TFBG humidity sensors with different diameters.

At a diameter of 20 μm, the sensitivity was −0.01 nm/% RH, and the linearity was 0.996. At a diameter of 35 μm, the sensitivity was −0.00425 nm/% RH, and the linearity was 0.990. At a diameter of 50 μm, the sensitivity was −0.0031 nm/% RH, and the linearity was 0.985. At a diameter of 55 μm, the sensitivity was −0.0022 nm/% RH, and the linearity was 0.995. At a diameter of 60 μm, the sensitivity was −0.0021 nm/% RH, and the linearity was 0.996. Through the experimental results detailed above, we found that the diameter of 20 μm had the best wavelength sensitivity, as shown in Figure 9. From the results for the above five diameters, it can be seen that the refractive index decreases when the humidity is increased from 20% RH to 80% RH. The fact that the wavelength is shifted from a long wavelength to a short wavelength can also be ascertained from Equation (4), so this paper selected a 20 μm diameter TFBG humidity sensor to undergo the whole humidification and dehumidification process.

### 4.4. Analysis and Discussion of Humidification and Dehumidification

**Figure 10:**
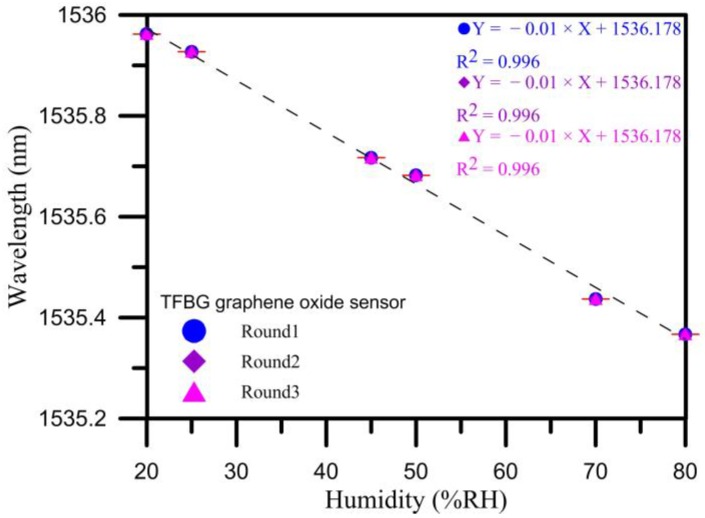
3-cycle repeat test TFBG humidity wavelength results.

**Figure 11:**
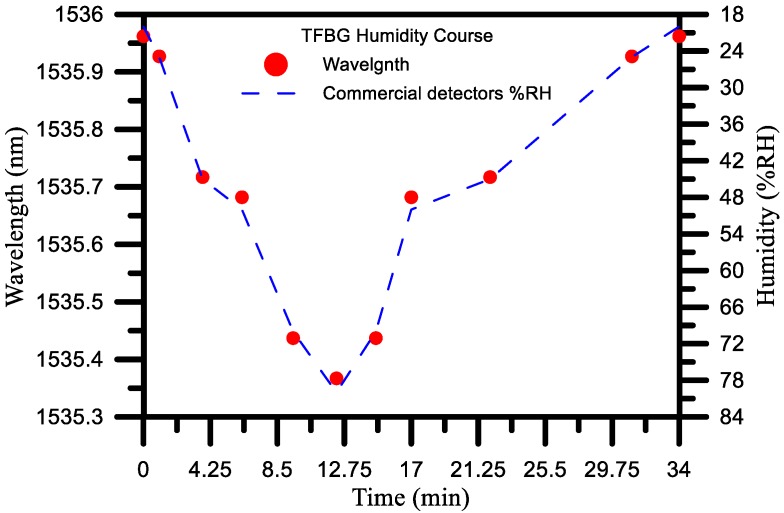
Response and recovery time of the TFBG sensor when subjected to a step humidity change from 20% to 80%.

The wavelength sensitivity in the first cycle was −0.01 nm/% RH, and the linearity was 0.996. The wavelength sensitivity in the second cycle was −0.01 nm/% RH, and the linearity was 0.996. The wavelength sensitivity in the third cycle was −0.01 nm/% RH, and the linearity was 0.996. The optimal wavelength sensitivity was −0.01 nm/% RH, and the linearity was 0.996. From the results for the three cycles, it can be seen that the sensitivity and linearity were the best in the third cycle. From the error bars shown in Figure 10 the average error of about 0.000001 can be obtained. According to the Figure 11, the response time for a humidity change from 20% to 80% RH was about 12.25 min, the recovery time was about 21.75 min. The results in Figure 11 indicate that the results for the humidification process exhibited very good reproducibility. This in turn shows that the graphene oxide film coating has good sensitivity and linearity when used with the TFBG sensor. 

After three cycles of testing, we can see that the relative humidity measured by the TFBG graphene oxide sensor was close to the relative humidity measured by the standard humidity sensor, which shows that the TFBG sensor with graphene oxide coating has the same accuracy for humidity sensing despite being cheaper.

From the analysis results detailed in Section 4.3, it can be seen that a diameter of 20 μm yielded the best humidity sensitivity, which is why this study subjected a 20 μm diameter humidity sensor to three cycles of testing in order to obtain an analysis of sensitivity, linearity, and standard deviations for the overall humidification and dehumidification process. As shown in Figure 11, as the humidity increased, the refractive index decreased, resulting in the wavelength being shifted from a long wavelength to a short wavelength. Specifically, the wavelength was 1535.962 nm when the humidity was 20%, whereas the wavelength was 1535.367 nm when the humidity was 80%. That is, as the humidity went from 20% RH to 80% RH, the total amount of change in the wavelength was 595 pm.

**Figure 12:**
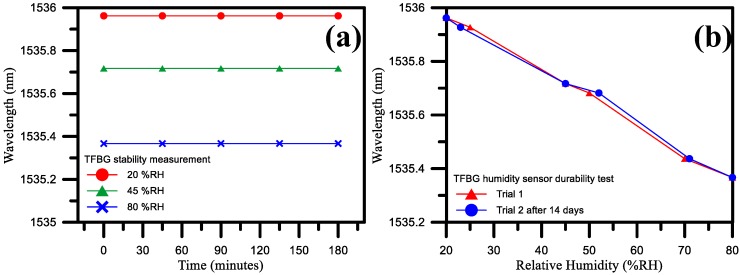
TFBG sensor stability and durability test results for (**a**) 20% RH, 45% RH, and 80% RH measurements; (**b**) trial 1 and trial 2 (conducted 14 days after trial 1).

We have provided the preliminary experimental data for a sensor stability and durability test. According to the experimental results, the humidity sensitivity measured at 14 days was still quite close to the initial sensitivity, as shown in Figure 12b. Furthermore, as shown in Figure 12a, the humidity measurement results for 20% RH, 45% RH, and 80% RH showed good stability. It is thus proven that the proposed sensor exhibits good stability and durability.

## 5. Conclusions

In this study, a TFBG humidity sensor was fabricated by coating TFBG with a graphene oxide film. The measurement results showed that the measurement sensitivity of the humidity was 4.695 times higher than 0.00213 nm/% RH [7], 5 times higher than 0.002 nm/% RH [8], 7.273 times higher than 0.001375 nm/% RH [13], and 2.625 times higher than 0.00381 nm/% RH [21], while the wavelength sensitivity was −0.01 nm/% RH and the linearity was 0.996. This optical fiber sensing system has high research and development value, as the TFBG can be used not only for relative humidity sensing, but also in temperature, strain, and magnetic field sensing applications, a versatility that suggests that its future is really limitless.
